# Adjunctive role of p16/Ki-67 dual-stain cytology in colposcopy referral pathways for cervical precancer and persistent high-risk HPV infection

**DOI:** 10.3389/fonc.2026.1794191

**Published:** 2026-07-13

**Authors:** Yingying Yue, Xuewang Guo, Yu Dou, Xiaowen Wu, Weina Yang, Yanying Xu

**Affiliations:** Department of Gynecology, The Second Hospital of Tianjin Medical University, Tianjin, China

**Keywords:** antigen Ki67, cervical cancer, HPV infection, p16 (INK4a), uterine cervical neoplasms

## Abstract

**Background:**

This study aimed to evaluate the adjunctive triage performance of p16/Ki-67 dual staining (DS) cytology for cervical lesions in a colposcopy-referred cohort, to analyze its correlation with lesion severity, and to explore its predictive value for persistent high-risk human papillomavirus (HR-HPV) infection.

**Methods:**

A total of 109 patients undergoing colposcopic cervical biopsy (recruited via standard HPV+TCT referral criteria) were included. We evaluated the incremental diagnostic performance of adding p16/Ki-67 dual-stain (DS) triage to the standard HPV+TCT referral workflow for detection of CIN2+ and CIN3 +. Among 66 HR-HPV-positive patients with CIN1 or lower lesions, p16/Ki-67 DS cytology was performed, and the patients were followed up for 6 months to assess the predictive value for persistent infection.

**Results:**

The positivity rate of p16/Ki-67 DS cytology gradually increased with the progression of cervical lesion severity (P<0.001). Further triage with p16/Ki-67 DS on the basis of combined HPV+TCT cervical cancer screening yielded sensitivity, specificity, accuracy and AUC of 77.8%, 88.6%, 84.4% and 0.822 ± 0.052 (95%CI: 0.721–0.923) for the diagnosis of CIN2+, and 93.3%, 81.3%, 81.7% and 0.823 ± 0.056 (95%CI: 0.713–0.933) for CIN3+, respectively. Among HR-HPV-positive CIN1/lower lesions, 87.1% of DS-positive patients had persistent infection compared to 62.9% of DS-negative patients (P = 0.025), with an odds ratio of 4.515 (P = 0.028).

**Conclusions:**

In this colposcopy-referred cohort defined by HPV and/or TCT-based referral criteria, p16/Ki-67 dual-stain (DS) cytology exhibited satisfactory adjunctive triage efficacy for identifying CIN2+ and CIN3+, and independently predicted persistent HR-HPV infection among patients with ≤CIN1 lesions. Notably, all analyses in this study are limited to diagnostic efficacy in this referral cohort; any potential clinical applications, including possible reductions in unnecessary procedures, remain exploratory hypotheses that require prospective clinical validation.

## Introduction

Cervical cancer (CC) is one of the most common malignant tumors in women worldwide and poses a serious threat to female health and safety (1). In developing countries, the incidence and mortality rates of CC are significantly higher, which is closely related to factors such as the coverage and rate of CC screening (2, 3). A core clinical challenge in gynecologic oncology is the accurate stratification of cervical lesions: most HR-HPV infections are transient and self-limited, while only a small subset of infections drive persistent cell cycle dysregulation and progress to clinically significant precancer or invasive disease (4, 5). Distinguishing biologically meaningful precancer with malignant transformation potential from benign reactive changes or transient HPV infection is critical to avoid both overtreatment of indolent lesions and missed diagnosis of progressive disease (6).

Current clinical evaluation of cervical lesions relies on HPV testing and ThinPrep cytologic test (TCT) as primary screening modalities (7). However, these methods have inherent limitations from an oncologic perspective: HPV testing alone cannot differentiate between transient infection and transforming infection that drives neoplastic progression, resulting in low specificity and substantial overtreatment. TCT is limited by inter-observer variability and cannot directly reflect the molecular alterations underlying malignant transformation (8). Neither modality can reliably predict which low-grade lesions will progress to high-grade precancer or cancer, creating an unmet need for biomarkers that directly indicate the oncogenic activity of HPV infection (9, 10). Additionally, existing screening strategies are insufficient to predict the persistence of HR-HPV infection in infected individuals.

P16/Ki-67 dual staining (DS) has emerged as a mechanistically relevant biomarker for cervical neoplasia, rather than merely an adjunct screening tool (11–16). p16 overexpression is a direct surrogate of HR-HPV E7 oncoprotein-mediated inactivation of the pRb tumor suppressor pathway, a hallmark event in cervical carcinogenesis. Ki-67 is a canonical marker of active cell proliferation (17, 18). In normal cervical epithelium, p16 and Ki-67 expression are mutually exclusive: p16-mediated cell cycle arrest would suppress Ki-67 expression. Co-expression of p16 and Ki-67 in the same cell therefore directly demonstrates cell cycle dysregulation, a defining feature of HPV-driven malignant transformation (19–22). This dual positivity identifies lesions with active oncogenic progression, rather than merely HPV exposure, making it a biologically meaningful marker of clinically significant cervical precancer.

As a non-invasive testing method, p16/Ki-67 DS cytology requires only exfoliated cervical cell samples and does not require morphological interpretation of positive cells, offering good reproducibility and operability (23–25). Currently, there is extensive research on the application of p16/Ki-67 dual-stain cytology in cervical cancer screening, primarily focusing on its triage efficacy under different cervical screening scenarios and its predictive role in cervical lesion recurrence. However, limited evidence is available regarding the incremental value of DS for risk stratification in colposcopy-referred patients, who represent the key population for clinical decision-making in precancer management.

The present study therefore aimed to: (1) evaluate the correlation between p16/Ki-67 DS positivity and histologic severity of cervical neoplasia; (2) assess the diagnostic performance of this marker as a triage biomarker for high-grade cervical precancerous lesions (CIN2+/CIN3+) in a colposcopy-referred cohort; (3) investigate its prognostic value for predicting persistent HR-HPV infection in patients with low-grade or benign lesions. This work seeks to provide translational evidence supporting p16/Ki-67 DS as an oncologic biomarker for precision risk stratification of cervical neoplasia.

## Materials and methods

### Study design and participants

A total of 109 patients were included in this study based on the inclusion and exclusion criteria. First, female patients scheduled for colposcopy and cervical biopsy at the Department of Gynecology, Second Hospital of Tianjin Medical University, from January 2024 to June 2024, were identified. Cervical exfoliated cell samples were collected from all patients, immediately placed in liquid-based cytology preservation solution, and processed within 1 h or stored at 2–8 °C and processed within one week. Patients with complete HPV, TCT, and pathological cervical biopsy results were included. All patients underwent HPV, TCT, and p16/Ki-67 DS testing prior to the colposcopic cervical biopsy. The relevant clinical information of the included patients was analyzed to statistically evaluate the diagnostic efficacy of the different screening methods for cervical lesions of varying grades.

Inclusion criteria included:scheduled colposcopy and simultaneous cervical biopsy, plus any one of: HPV16/18 positivity; other HR-HPV infection with ASC-US or higher TCT result; or LSIL or higher TCT result. Exclusion criteria included: prior cervical disease treatment, pregnancy/lactation, other concurrent malignancies, prior total/subtotal hysterectomy, or acute reproductive system inflammation.

Patients pathologically diagnosed with cervical intraepithelial neoplasia grade 1 or lower (≤CIN1), and positive for HR-HPV were included in subsequent study, while patients who received cervical physical therapy or anti-HPV treatment within 6 months before enrollment or during the 6-month follow-up period were excluded(n=66). All included patients underwent outpatient follow-up examination 6 months later. Persistent HR-HPV infection was defined as positive HR-HPV at the initial re-examination and persistent positivity for the same HR-HPV type at the follow-up re-examination 6 months later. During the follow-up, the following examination results and relevant clinical data were collected: medical history, gynecological examination, HPV test results, cervical biopsy pathological records, and general clinical data, including age, presence of vaginitis, oral intake of short-acting contraceptives, menopausal status, diabetes mellitus, and hypertension. The correlation between relevant indicators and persistent HR-HPV infection was also analyzed.

This study was approved by the Ethics Committee of the Second Hospital of Tianjin Medical University (approval no. KY2025K118). All procedures performed in this study were in accordance with the ethical standards of the institutional research committee and with the 1964 Helsinki Declaration and its later amendments or comparable ethical standards. Written informed consent was obtained from all individual participants included in the study.

### Collection and preparation of cervical exfoliated cell samples

The patients were placed in the dorsal lithotomy position on a gynecological examination table. The external genitalia were routinely disinfected, and a sterile speculum was inserted into the vagina to fully expose the cervix. Excess mucus was gently removed using sterile cotton balls. The tip of the cervical brush was gently inserted into the cervical canal, and the brush was rotated clockwise 5–10 times around the external cervix to ensure adequate collection of cervical epithelial cells. The brush was then immersed in a cell preservation solution. After 1 h, a DC-4212 integrated slide-making and staining machine was used to prepare 13-mm-diameter cytological smears using the liquid-based cytology technique, with each smear requiring at least 5,000 squamous cells. The smears were fixed with 95% ethanol for 30 min and soaked in distilled water for further use.

### p16/Ki-67 cytological dual staining

Cervical cell smears were placed in immunohistochemical antigen retrieval buffer and heated in a pressure cooker at 1600 W and 210 °C for antigen fixation. After fixation, the smears were removed, and 50 µL of peroxidase blocking agent was added dropwise and incubated at room temperature for 10 minutes. Sequentially, 50 µL of p16/Ki-67 antibody (Guangzhou AnB Ping Medical Technology Co., Ltd.) and 50 µL of polymer secondary antibody reagent were added dropwise and incubated at room temperature for 45 minutes, followed by washing with TBS buffer. The smears were incubated with the prepared 3,3’-diaminobenzidine chromogenic solution at room temperature for 5 minutes, then 100 µL of freshly prepared Fast-Red chromogenic solution was added dropwise and incubated in the dark at room temperature for 15 minutes. Counterstaining was performed with hematoxylin for 30–60 s, followed by thorough rinsing with distilled water, ethanol gradient dehydration, xylene clearing, and cover slipping with a neutral gum. The stained slides were observed and interpreted using an optical microscope.

### Result interpretation

**Figure 1:**
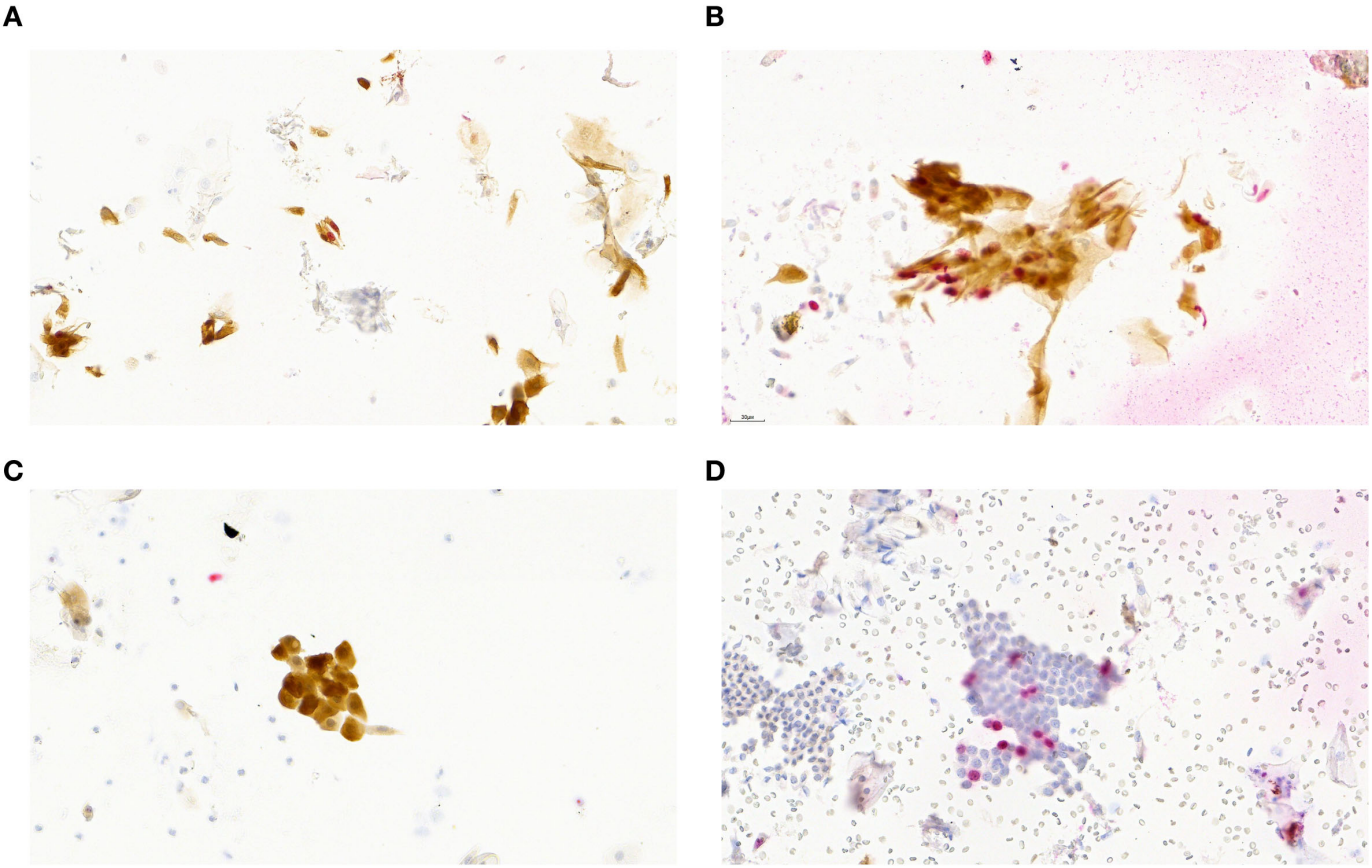
Typical images of different results for p16/Ki−67 dual staining. (a) P16/Ki67 DS+, (b) P16/Ki67 DS+, (c) P16+/Ki67-, (d) P16-/Ki67+

In interpreting the staining results, a cell was considered positive when it exhibited nuclear red stain for ki67 with concurrent cytoplasmic brown stain for P16. Both nuclear red and cytoplasmic brown staining must be visualized simultaneously in the same focal plane. The presence of ≥1 dual-stained positive cell in the smear was interpreted as a positive result (**Figure 1**). Result evaluation was independent of the cellular morphology (26, 27). The staining results were evaluated by two pathologists from the Second Hospital of Tianjin Medical University under double-blind conditions. To evaluate the reproducibility of DS staining interpretation, two pathologists independently scored all specimens without knowledge of the clinical data. Interobserver agreement was analyzed using Cohen’s kappa coefficient. A discrepant case was resolved by consensus discussion.

### Statistical analysis

Data analysis was performed using SPSS 24.0 (IBM Corp., Armonk, NY, USA) with partial assistance from R 4.4.1 (R Project for Statistical Computing, Vienna, Austria). Categorical data are presented as frequency (n) and percentage (%). Fisher tests were used to compare the rates between groups to analyze the screening performance of p16/Ki-67 DS cytology in different cervical lesions and its association with lesion severity. The incremental diagnostic performance of adding DS to the standard HPV+TCT referral workflow was evaluated by calculating sensitivity, specificity, and accuracy. Positive TCT results were defined as ASC-US or higher. Positive HPV were defined if any HR-HPV was positive. McNemar’s test was used to compare differences between screening methods. Receiver operating characteristic (ROC) curves were plotted to calculate the area under the curve (AUC) and 95% confidence interval (CI), and DeLong’s test was used to compare statistical differences in AUC to assess the diagnostic efficacy of each method for CIN2+ and CIN3 +. Additionally, based on the existing colposcopy referral strategy, p16/Ki-67 DS was introduced as an auxiliary triage strategy, and McNemar’s test was used to evaluate differences in diagnostic performance between the standard workflow and DS-augmented workflow. All tests were two-sided, and statistical significance was defined as P < 0.05. Categorical data were described using frequency (n) and percentage (%), with the chi-square test or Fisher’s exact test for intergroup comparisons. Variables with P < 0.1 in univariate comparisons were further analyzed using multivariate logistic regression. Statistical significance was set at P < 0.05.

## Results

### Correlation between p16/Ki-67 DS positivity and histologic severity of cervical neoplasia

**Figure 2:**
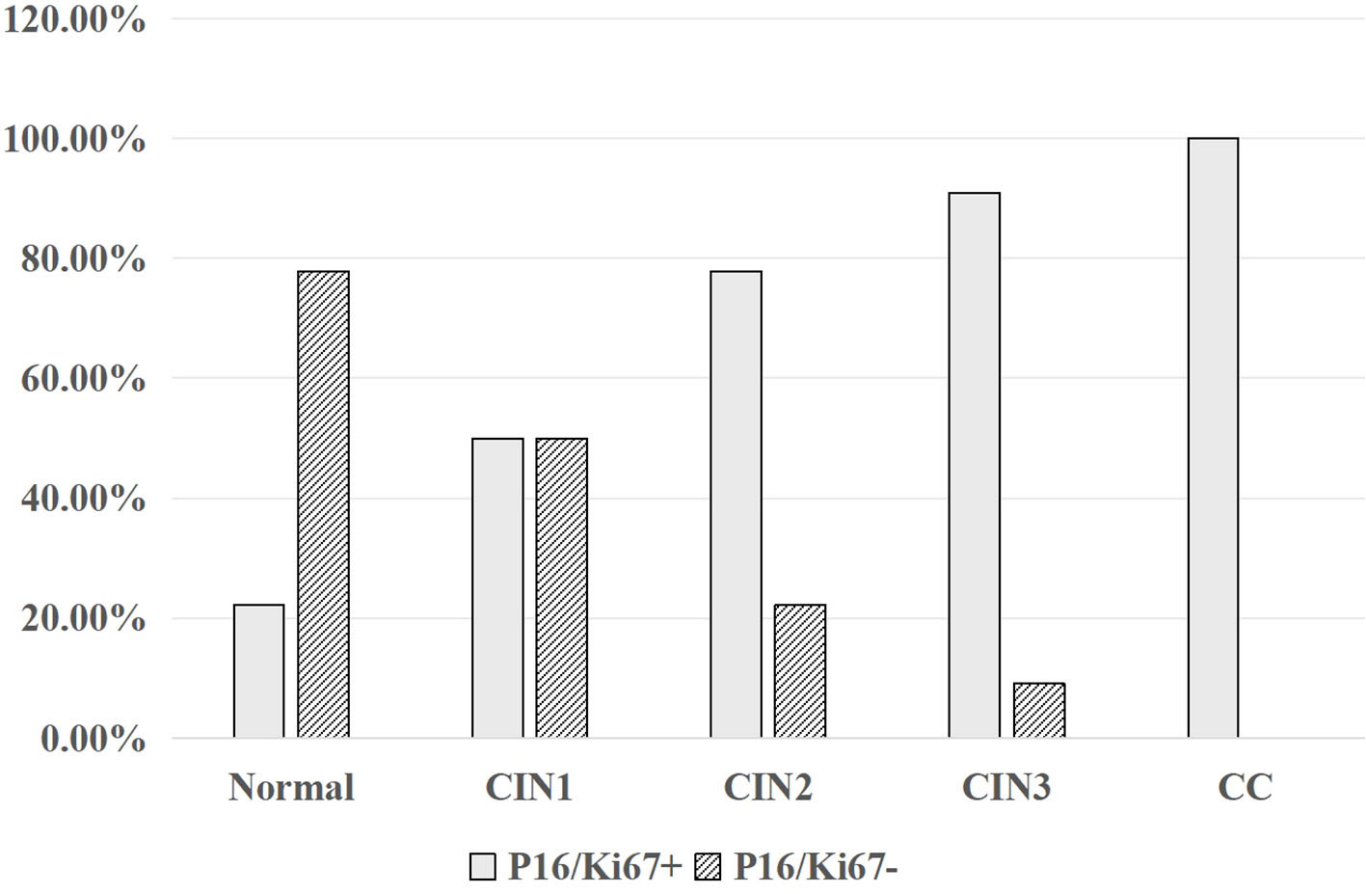
p16/Ki-67 DS in different cervical lesion. CC, cervical carcinoma; CIN, cervical intraepithelial neoplasia; DS, dual-stain.

The interobserver agreement for DS staining interpretation was excellent (kappa = 0.98). An escalating trend in HPV infection was observed with the progression of cervical lesions, similarly, analysis of the TCT results showed a gradual worsening of TCT outcomes with increasing severity of cervical lesions, and there were statistically significant differences in two groups (P=<0.001;P=<0.001) (**Table 1**). The results of p16/Ki-67 DS demonstrated a stepwise increase in positive rates with worsening of cervical lesions. The normal group exhibited the lowest positivity rate for p16/Ki-67 DS (22.22%, 16/72), whereas the positivity rates in the CIN1, CIN2, and CIN3 groups were 50.00% (5/10), 77.78% (7/9), and 90.91% (10/11), respectively. The positivity rate in the CC group was 100.00% (7/7). There was a statistically significant increase in the p16/Ki-67 DS-positive rates with disease progression (χ²=35.937, P < 0.001), indicating a correlation between the p16/Ki-67 DS-positive rates and the severity of cervical lesions (**Table 1** and **Figure 2**).

**Table 1:** HPV, TCT and p16/Ki-67 DS in different cervical lesions.

Pathological results							Total	χ²	P
Test item		Normal(n,%)	CIN1(n,%)	CIN2(n,%)	CIN3(n,%)	CC(n,%)			
TCT	NILM	23(31.9%)	3(30.0%)	2(22.2%)	2(18.2%)	0(0.0%)	30(27.5%)	46.127	<0.001
	ASCUS	33(45.8%)	1(10.0%)	3(33.3%)	4(36.4%)	1(14.3%)	42(38.5%)		
	ASC-H	0(0.0%)	1(10.0%)	0(0.0%)	2(18.2%)	1(14.3%)	4(3.7%)		
	LSIL	14(19.4%)	5(50.0%)	4(44.4%)	0(0.0%)	2(28.6%)	25(22.9%)		
	HSIL	2(2.8%)	0(0.0%)	0(0.0%)	3(27.3%)	3(42.9%)	8(7.3%)		
HPV	HPV 16/18	14(19.4%)	4(40.0%)	4(44.4%)	7(63.6%)	5(71.4%)	34(31.2%)	42.385	<0.001
	Other HR-HPV	47(65.3%)	6(60.0%)	4(44.4%)	4(36.4%)	2(28.6%)	63(57.8%)		
	HR-HPV	61(84.7%)	10(100.0%)	8(88.9%)	11(100.0%)	7(100.0%)	97(89.0%)		
	LR-HPV/Negative	11(15.3%)	0(0.0%)	1(11.1%)	0(0.0%)	0(0.0%)	12(11.0%)		
p16/Ki-67 DS	Positive	16(22.2%)	5(50.0%)	7(77.8%)	10(90.9%)	7(100.0%)	45(41.3%)	68.734	<0.001
	Negative	56(77.8%)	5(50.0%)	2(22.2%)	1(9.1%)	0(0.0%)	64(58.7%)		

### Performance of p16/Ki-67 DS cytology as an adjunctive triage biomarker for CIN2+ and CIN3+

**Figure 3:**
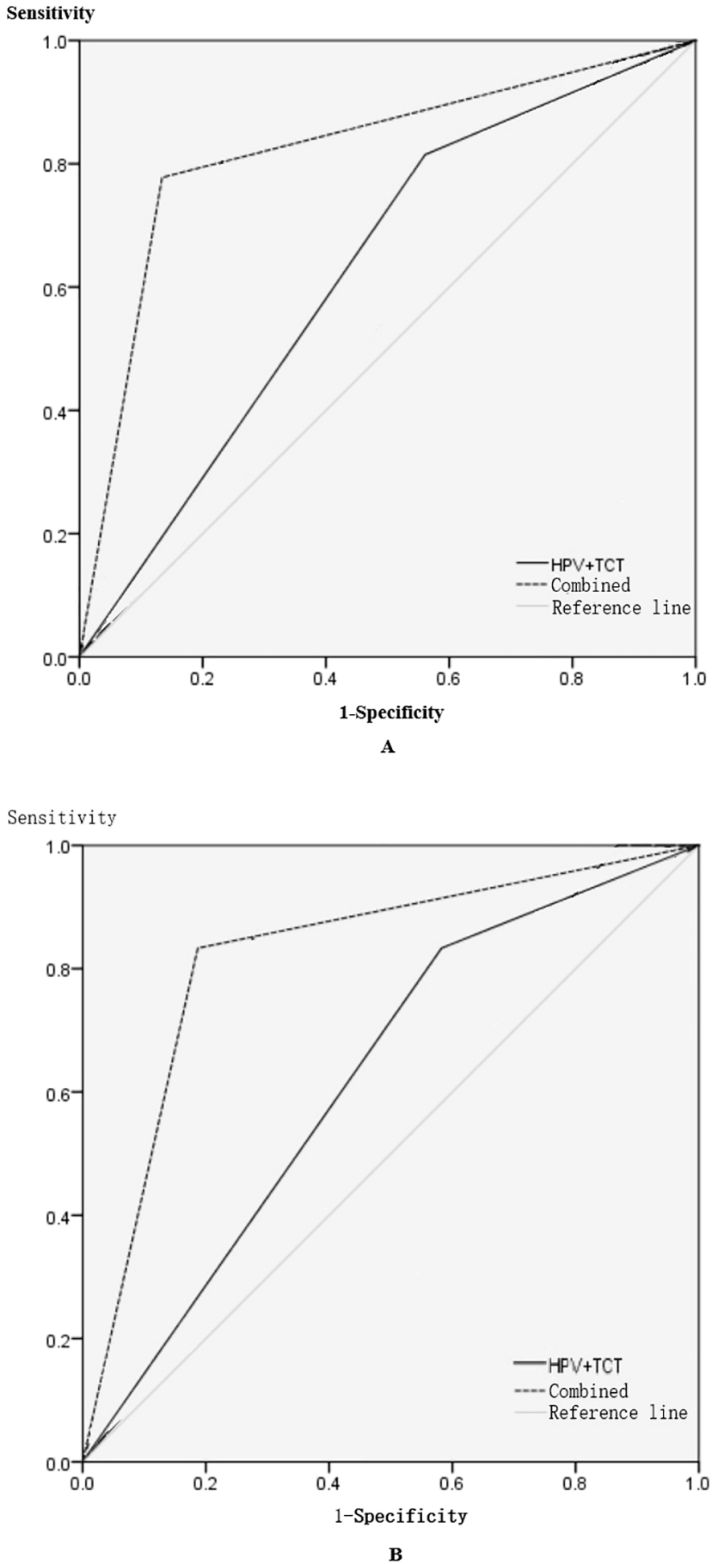
ROC curve of HPV, TCT, p16/ki-67 DS detected alone and in combination. **(A)** Pathological Classification is CIN2 +. **(B)** Pathological Classification is CIN3 +. DS, dual-stain; HPV, human papillomavirus; TCT, ThinPrep cytologic test.

We evaluated the diagnostic performance of p16/Ki-67 DS when used as an adjunctive triage step within the standard HPV+TCT combined colposcopy referral workflow (the clinical standard for patient enrollment in this study), for detection of histologically confirmed high-grade cervical precancer. For identification of CIN2+, the standard HPV+TCT referral workflow yielded a sensitivity of 81.5%, specificity of 43.9%, and accuracy of 53.2%, with an area under the receiver operating characteristic curve (AUC) of 0.627 ± 0.059 (95% CI: 0.511–0.743). When adding p16/Ki-67 DS as an adjunctive triage step in this diagnostic study, the workflow maintained high sensitivity of 77.8%, specificity of 86.6% and overall accuracy of 84.4% at the diagnostic level, corresponding to an AUC of 0.822 ± 0.052 (95% CI: 0.721–0.923, P<0.001 relative to the standard workflow alone). For identification of CIN3+, the standard HPV+TCT workflow yielded a sensitivity of 83.3%, specificity of 41.8%, and accuracy of 48.6%, with an AUC of 0.625 ± 0.067 (95% CI: 0.494–0.756). Augmentation with p16/Ki-67 DS triage preserved sensitivity for CIN3+ at 83.3% while maintaining specificity of 81.3% and accuracy of 81.7%, corresponding to an AUC of 0.823 ± 0.056 (95% CI: 0.713–0.933, P<0.001 relative to the standard workflow alone). All performance data reflect the incremental diagnostic value of adding DS to the existing standard clinical pathway within this referral-enriched cohort (**Table 2** and **Figure 3**).

**Table 2:** Incremental diagnostic value of adding p16/Ki-67 dual-stain triage to standard HPV+TCT colposcopy referral strategy for identification of CIN2+ and CIN3+.

Pathological classification	Screening test	Sensitivity(%)	Specificity(%)	Accuracy(%)	Youden index(%)	AUC (95% Confidence Interval)	P(AUC)
CIN2+	HPV+TCT	81.5	43.9	53.2	25.4	0.627 ± 0.059 ([0.511, 0.743])	<0.001
	Combination	77.8	86.6	84.4	64.4	0.822 ± 0.052 ([0.721, 0.923])	
CIN3+	HPV+TCT	83.3	41.8	48.6	25.1	0.625 ± 0.067 ([0.494, 0.756])	<0.001
	Combination	83.3	81.3	81.7	64.6	0.823 ± 0.056 ([0.713, 0.933])	

### Analysis of incorporating p16/Ki-67 DS cytology into colposcopy referral strategies

p16/Ki-67 DS cytology was introduced as an auxiliary triage strategy into three existing colposcopy referral criteria: a. HPV16/18 group (HPV16 and/or HPV18-positive), b. other HR-HPV-positive (excluding 16/18) with TCT results of atypical squamous cells of undetermined significance (ASC-US) or higher, and c. TCT result of LSIL or higher, forming. When DS was added as an adjunctive test (groups d, e, and f), the DS-augmented groups showed comparable diagnostic sensitivity and higher diagnostic specificity and accuracy for CIN2+ detection relative to the conventional referral groups (groups a, b, and c) in this cohort. These performance differences are observed within the context of our retrospective study design and do not constitute evidence of real-world clinical benefit. Specifically, in the HPV16/18-positive group, the addition of DS increased specificity from 78.0% to 90.2% (P<0.01) and accuracy from 73.4% to 81.7% (P<0.001). Similar improvements in specificity and accuracy were observed in the other HR-HPV with ASC-US+ group (specificity: 47.6% to 89.0%, P<0.001; accuracy: 44.0% to 74.3%, P<0.001) and the LSIL+ group (specificity: 73.2% to 89.0%, P<0.001; accuracy: 68.8% to 78.9%, P<0.001), with only modest reductions in sensitivity (all P>0.05). (**Table 3**).

**Table 3:** Adjunctive diagnostic value of p16/Ki-67 DS [n=(%)].

Groups	Strategy	Sensitivity	Specificity	Accuracy	P
a	Conventional	59.3	78.0	73.4	P_Se_=1.000 P_Sp_<0.05 P_Ac_<0.001
d	Conventional + DS (adjunctive)	55.6	90.2	81.7	
b	Conventional	33.3	47.6	44.0	P_Se_=1.000 P_Sp_<0.001 P_Ac_<0.001
e	Conventional + DS (adjunctive)	29.6	89.0	74.3	
c	Conventional	55.6	73.2	68.8	P_Se_=0.618 P_Sp_<0.001 P_Ac_<0.001
f	Conventional + DS (adjunctive)	48.1	89.0	78.9	

a. HPV16/18(HPV16 and/or HPV18) d.HPV16/18+p16/Ki-67 DS.

b: Other HR-HPV-positive (excluding 16/18) with TCT results of ASC-US or higher e: Other HR-HPV-positive (excluding 16/18) + ASC-US+ p16/Ki-67 DS.

c. TCT result of LSIL or higher f.TCT result of LSIL or higher+p16/Ki-67 DS.

P_Se_: P value of Sensitivity; P_Sp_: P value of Specificity; P_Ac_: P value of Accuracy.

### Analysis of risk factors for persistent HR-HPV infection in populations with CIN1 and lower-grade lesions

Univariate analysis showed statistically significant differences in the positive rate of p16/Ki-67 DS cytology testing and age between the two groups (P<0.05), which were factors influencing persistent HR-HPV infection in CIN1 and lower-grade lesions. Other variables, such as oral short-acting contraceptive use, menopausal status, hypertension, and diabetes, showed no statistically significant differences (P>0.05) (**Table 4A**). Variables with P<0.1 in the univariate analysis were included in the multivariate logistic regression model for analysis. The results showed that positive p16/Ki-67 DS cytology testing was an independent risk factor for persistent HR-HPV infection (P<0.05), whereas oral short-acting contraceptive use and menopausal status did not significantly correlate with persistent HR-HPV infection (P>0.05) (**Table 4B**).

**Table 4A:** comparison of clinical parameters of 66 high-risk HPV patients [n(%)].

Clinical parameters	Persistent infection n=49	Transient infection n=17	Statistic	P
Age(Years)	47.49 ± 14.343	39.76 ± 10.347	t=2.040	0.046
HPV				
16/18	12(24.5)	4(23.5)	χ²=0.006	0.608
Other HR-HPV	37(75.5)	13(76.5)		
p16/Ki-67 DS				
Positive	27(55.1)	4(23.5)	χ²=5.051	0.025
Negative	22(44.9)	13(76.5)		
Vaginitis				
Positive	16(32.7)	7(41.2)	χ²=0.404	0.525
Negative	33(67.3)	10(58.8)		
Short-acting Contraceptive				
Yes	3(6.1)	4(23.5)	χ²=3.520	0.066
No	46(93.9)	13(76.5)		
Menopause				
Yes	21(42.9)	3(17.6)	χ²=3.466	0.063
No	28(57.1)	14(82.4)		
hypertension				
Yes	5(10.2)	2(11.8)	χ²=0.032	1.000
No	44(89.8)	15(88.2)		
Diabetes				
Yes	5(10.2)	1(5.9)	χ²=0.281	1.000
No	44(89.8)	16(94.1)		
Pathology				
Normal	8(16.3)	16(94.1)	χ²=1.151	0.427
CIN1	41(83.7)	1(5.9)		

**Table 4B:** Multivariate logistic regression analysis between variables and persistent high-risk HPV infection.

Clinical parameters	β	Standard error	Wald	*P*	OR	95%CI
p16/Ki-67 DS	1.510	0.668	4.818	0.028	4.515	1.176∼17.420
Menopause	1.187	1.157	0.026	0.872	1.205	0.125∼11.631
Short-acting Contraceptive	-1.026	0.893	1.319	0.251	0.358	0.062∼2.064
Age(Years)	0.045	0.040	1.238	0.266	1.046	0.966∼1.132

## Discussion

CC is one of the most common malignant tumors in women worldwide, with over 500,000 new cases and 270,000 deaths annually owing to late diagnosis or inadequate treatment (28–30). However, CC is preventable through early screening, as its progression is typically gradual, evolving from CIN1 to CIN2+ and eventually to invasive cancer over years or even decades (31). Current primary screening methods have notable limitations in specificity and in predicting persistent HR-HPV infection. p16/Ki-67 dual-stain (DS) cytology has emerged as a promising biomarker that can help identify true transforming infections by detecting cell cycle dysregulation (32, 33).

P16 is a key negative regulator of the cell cycle. Inhibition of cyclin-dependent kinases (CDK4/6) prevents cells from transitioning from the G1 to S phase, thereby suppressing cell proliferation. As a marker of cell proliferation, Ki67 is expressed exclusively in actively dividing cells (34–36). The combined use of these two markers helps identify lesions at risk of malignant progression. p16/Ki-67 DS combines the expression patterns of these two proteins to identify CIN2+ and distinguish benign HPV-induced changes from true precancerous lesions (37, 38). In recent years, both the 2024 American Society for Colposcopy and Cervical Pathology guidelines and the Chinese expert consensus have recommended p16/Ki-67 DS cytology as an adjuvant biomarker for cervical precancer identification, primarily to improve diagnostic specificity in triage settings (13, 24). From an oncologic and translational perspective, the core value of p16/Ki-67 dual staining extends far beyond screening performance optimization. Co-expression of p16 and Ki-67 directly reflects HR-HPV-mediated cell cycle dysregulation: p16 overexpression indicates disruption of the pRb tumor suppressor pathway by HPV E7 oncoprotein, while concurrent Ki-67 positivity confirms uncontrolled cell proliferation. This dual positivity is a specific biomarker of biologically meaningful cervical precancer with true malignant transformation potential, rather than transient HPV infection or benign reactive changes. The results of this study confirmed that the positive rate of p16/Ki-67 DS cytology was the lowest in the normal group (22.22%), while the positive rates in the CIN1, CIN2, CIN3, and CC groups were 50.00%, 77.78%, 90.91%, and 100.00%, respectively. There was a statistically significant difference in p16/Ki-67 DS expression among the four groups (χ²=68.734, P<0.001), indicating that p16/Ki-67 DS shows promising diagnostic value for identifying high-grade cervical lesions. Our findings that DS positivity correlates strongly with histologic severity and independently predicts persistent HR-HPV infection further support its role as a prognostic biomarker for cervical neoplasia progression. This oncologic significance, rather than incremental improvements in screening metrics, represents the primary translational value of our findings.

**Figure 4:**
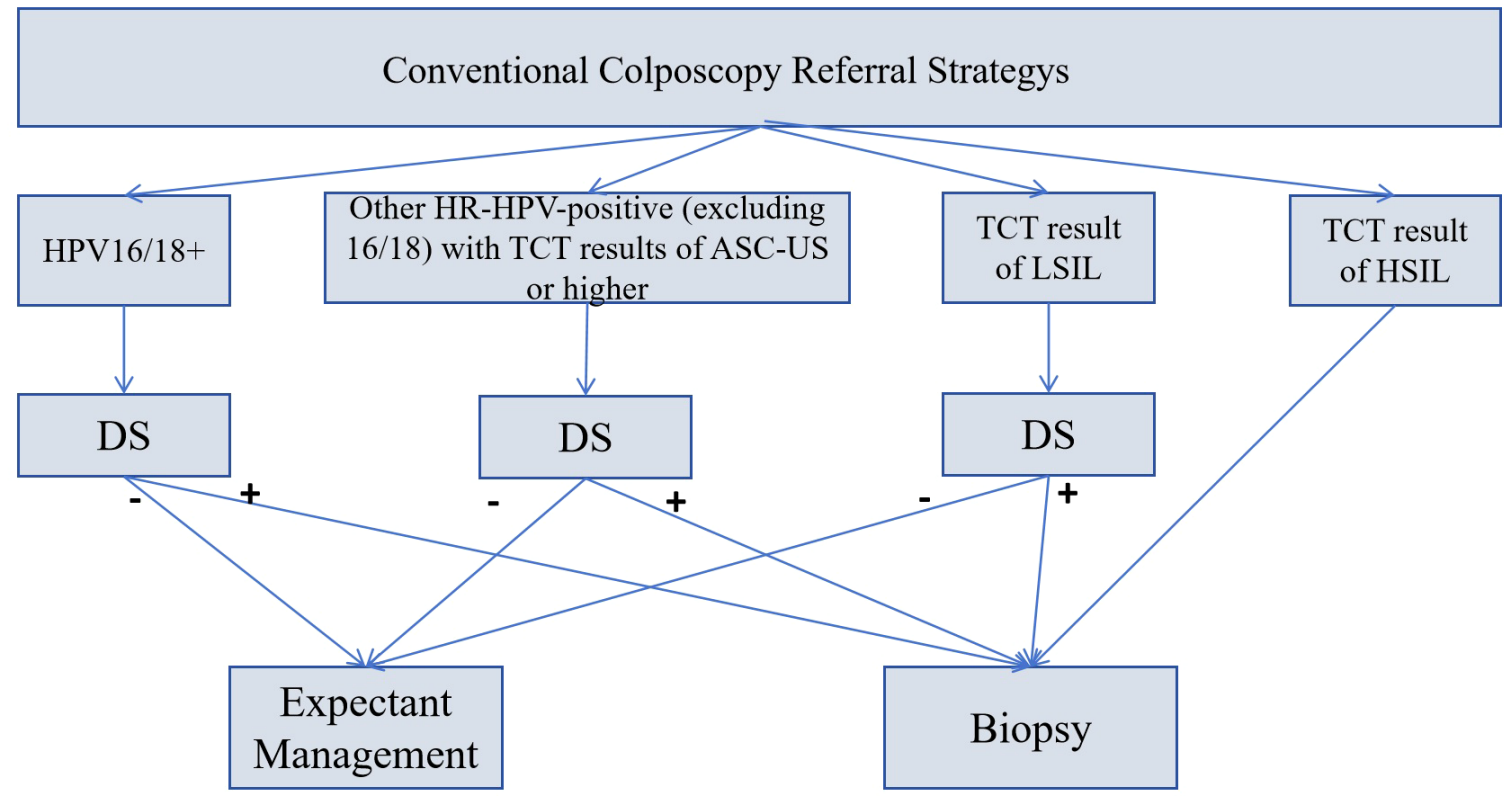
Proposed adjunctive triage algorithm incorporating p16/Ki-67 DS cytology into conventional colposcopy referral strategies.

Based on our retrospective dataset of 109 patients, we propose a hypothesis-generating triage framework incorporating p16/Ki-67 DS as an adjunctive marker (**Figure 4**). In this exploratory framework, patients with TCT HSIL would proceed directly to biopsy, while p16/Ki-67 DS would be used to guide further management for all other cases (TCT < HSIL), with biopsy considered only for DS-positive patients. Performance estimates from our cohort suggest this framework could maintain high sensitivity for clinically significant lesions while improving specificity: the overall sensitivity of DS for CIN2+ was 88.9% (24/27), and only 1 of 3 DS-negative CIN2+ cases would not be captured by the HSIL direct biopsy rule. Importantly, this framework is not a clinically validated management strategy: it is derived exclusively from a small, retrospective, referral-enriched cohort, and all estimates of potential biopsy reduction represent theoretical projections. This framework requires large-scale prospective validation to confirm its safety, efficacy, and clinical utility before any real-world implementation can be considered.

Persistent HR-HPV infection is a major risk factor for the development and progression of CC (39–41). Some HPV infections can be cleared by the immune system, and persistent infections are associated with a higher risk of precancerous lesions and carcinogenesis (42, 43). Identifying high-risk populations with persistent HR-HPV infection as early as possible and implementing precision screening and long-term management are crucial for reducing missed diagnosis rates and controlling the incidence of CC (44, 45). For individuals with HR-HPV infection but no cytological abnormalities, a negative p16/Ki-67 DS result may indicate a lower risk of persistent infection and potential for closer follow-up. Whether this could reduce unnecessary colposcopy referrals remains an unproven hypothesis requiring prospective clinical validation.

Although many studies have been conducted on HPV infection, few have predicted persistent HR-HPV infection, and there is still a lack of effective detection tools with limited guidance for the management of HPV infection in different situations (46). Currently, no reports exist on the correlation between p16/Ki-67 DS and persistent HR- HPV infection. This study found that in untreated patients with HR-HPV- with CIN1 and lower-grade lesions, positivity for p16/Ki-67 DS cytology was significantly associated with persistent HR-HPV infection (OR = 4.515, 95% CI = 1.176–17.420, P = 0.028). Among them, 87.1% (27/31) of the DS-positive patients remained HR-HPV-positive after six months of follow-up, whereas only 12.9% (4/31) cleared the infection, suggesting that p16/Ki-67 DS positivity reflects cell cycle dysregulation and cellular dysfunction caused by persistent infection. Mechanistically, the E7 protein of HR-HPV induces p16 overexpression by phosphorylating RB proteins, leading to loss of cell cycle control (47). High Ki-67 expression reflects active cell proliferation (48). Under normal conditions, p16 and Ki-67 are not co-expressed in the same cells, and p16/Ki-67 DS positivity indicates cell cycle disruption (49). The findings of this study further support this mechanism, demonstrating that p16/Ki-67 DS cytology, as a diagnostic marker of cellular lesions, shows promise as a candidate predictor of persistent HR-HPV infection. This provides exploratory biological evidence for future research into optimized management of HPV-positive individuals, but does not constitute evidence of clinical benefit.

In this diagnostic efficacy study, p16/Ki-67 DS enables further molecular risk stratification of referred patients at the diagnostic level. Diagnostic performance analyses based on 109 patients in this referral cohort show that adding DS as an adjunctive triage step yields higher diagnostic specificity for high-grade cervical lesions while preserving high diagnostic sensitivity. Whether this diagnostic performance translates to reduced unnecessary procedures or improved clinical management remains unproven, and represents an exploratory hypothesis for future prospective research. Meanwhile, p16/Ki-67 DS shows promise as a candidate biomarker for predicting persistent high-risk HPV infection, which also requires further clinical validation. This study has several important limitations that must be acknowledged. First, the sample size is relatively small (n=109), and all participants were colposcopy-referred patients selected based on HPV and/or TCT abnormalities. This referral-enriched design means: (1) all performance comparisons between DS and HPV/TCT are not fully independent, and results cannot be extrapolated to general population screening; (2) any proposed management frameworks derived from this cohort are inherently hypothesis-generating and require prospective validation. Second, this is a retrospective diagnostic efficacy study that did not evaluate clinical outcomes, referral volume reduction, cost-effectiveness, or real-world implementation feasibility. No claims regarding clinical benefit or optimization of referral pathways can be made based on these data alone. Future large-scale, multicenter, prospective studies are needed to validate the diagnostic and prognostic value of p16/Ki-67 DS, and to explore whether this biomarker can be integrated into clinical management pathways to deliver measurable patient benefits. In addition, further training and experience for physicians are needed to improve the consistency of the p16/ki-67 DS cytological test (50). P16/Ki-67 DS reflects cell cycle dysregulation, which can be influenced by multiple factors including inflammatory stimuli, potentially causing interference with result interpretation. Nevertheless, like many other molecular biomarkers, p16/Ki-67 DS holds exploratory potential as an auxiliary indicator for diagnostic risk stratification, and its clinical benefit requires further validation in prospective studies.

## Conclusions

In this colposcopy-referred cohort defined by HPV and/or TCT referral criteria, p16/Ki-67 dual-stain (DS) cytology demonstrated favorable adjunctive triage diagnostic performance for CIN2+ and CIN3+ detection, and was an independent predictor of persistent HR-HPV infection in patients with ≤CIN1 lesions. Diagnostic performance analyses show differences in diagnostic specificity and sensitivity between the standard workflow and DS-augmented workflow for high-grade lesions, with the latter showing higher specificity and comparable sensitivity. All findings should be interpreted within the context of the retrospective, referral-enriched study design, and potential clinical applications remain exploratory hypotheses requiring prospective validation.

## Data Availability

The original contributions presented in the study are included in the article/supplementary material. Further inquiries can be directed to the corresponding author.
